# A 2 miRNAs-based signature for the diagnosis of atherosclerosis

**DOI:** 10.1186/s12872-021-01960-4

**Published:** 2021-03-24

**Authors:** Xiujiang Han, Huimin Wang, Yongjian Li, Lina Liu, Sheng Gao

**Affiliations:** 1 Department of Geriatrics, Tianjin NanKai Hospital, No. 6 Changjiang Road, Nankai District, Tianjin City, 300100 China; 2 Department of Neurology, Tianjin NanKai Hospital, Tianjin City, 300100 China; 3 First Department of Cardiovascular Medicine, Tianjin NanKai Hospital, Tianjin City, 300100 China; 4 Nankai University, No. 94 Weijin Road, Nankai District, Tianjin City, 300071 China

**Keywords:** Atherosclerosis, Diagnosis, miRNAs, Signature, WGCNA, Logistic regression model

## Abstract

**Background:**

Atherosclerosis (AS) is a leading cause of vascular disease worldwide. MicroRNAs (miRNAs) play an essential role in the development of AS. However, the miRNAs-based biomarkers for the diagnosis of AS are still limited. Here, we aimed to identify the miRNAs significantly related to AS and construct the predicting model based on these miRNAs for distinguishing the AS patients from healthy cases.

**Methods:**

The miRNA and mRNA expression microarray data of blood samples from patients with AS and healthy cases were obtained from the GSE59421 and GSE20129 of Gene Expression Omnibus (GEO) database, respectively. Weighted Gene Co-expression Network Analysis (WGCNA) was performed to evaluate the correlation of the miRNAs and mRNAs with AS and identify the miRNAs and mRNAs significantly associated with AS. The potentially critical miRNAs were further optimized by functional enrichment analysis. The logistic regression models were constructed based on these optimized miRNAs and validated by threefold cross-validation method.

**Results:**

WGCNA revealed 42 miRNAs and 532 genes significantly correlated with AS. Functional enrichment analysis identified 12 crucial miRNAs in patients with AS. Moreover, 6 miRNAs among the identified 12 miRNAs, were selected using a stepwise regression model, in which four miRNAs, including hsa-miR-654-5p, hsa-miR-409-3p, hsa-miR-485-5p and hsa-miR-654-3p, were further identified through multivariate regression analysis. The threefold cross-validation method showed that the AUC of logistic regression model based on the four miRNAs was 0.7308, 0.8258, and 0.7483, respectively, with an average AUC of 0.7683.

**Conclusion:**

We identified a total of four miRNAs, including hsa-miR-654-5p and hsa-miR-409-3p, are identified as the potentially critical biomarkers for AS. The logistic regression model based on the identified 2 miRNAs could reliably distinguish the patients with AS from normal cases.

**Supplementary Information:**

The online version contains supplementary material available at 10.1186/s12872-021-01960-4.

## Highlights

- A total of 2 miRNAs, including hsa-miR-654-5p and hsa-miR-409-3p, are identified as the potentially critical biomarkers for atherosclerosis;
- The logistic regression model based on the identified 2 miRNAs, including hsa-miR-654-5p and hsa-miR-409-3p, could reliably distinguish the atherosclerosis patients from normal cases.

## Introduction

Atherosclerosis (AS) is a chronic arterial disorder and a significant determinant of vascular death [1]. Fatty streaks in arterial walls regularly develop into characteristic plaques and atheroma [2]. The acute rupture of these atheromatous plaques leads to local thrombosis, causing partial or total occlusion of the affected artery [3]. AS is featured by the progressive accumulation of lipids in the intimal space of the atrial walls, which results in several complications, such as oxidative stress, endothelial dysfunction, and chronic low-grade inflammation [4]. AS serves as an inflammatory disease that involves the accumulation of fatty components and fibrous in the intima of medium and large arteries such as the peripheral artery, carotid artery, and coronary artery, and the clinical manifestations vary with the arteries induced [5, 6]. AS are still the leading cause of death and loss of productive life years globally, although considerable advances in diagnosis, prevention, and therapy have been made [7]. Moreover, due to the early symptoms of AS are not obvious or even asymptomatic, early detection and early intervention can prevent the disease from continuing to develop in a more serious direction, which is extremely critical for the treatment of the disease [8]. Consequently, there is an increased need to identify the innovative biomarkers and predicting models for the diagnosis of AS.

Aberrantly expressed genes may be served as the potential diagnostic biomarkers of AS. For instance, an analysis of gene expression profiling identifies APH1B, JAM3, FBLN2, CSAD and PSTPIP2 as the potential diagnostic biomarkers for AS [9]. Intercellular adhesion molecule-1 expression and serum level could serve as diagnostic markers of pre-clinical AS [10]. Meanwhile, it has been recognized that combining various biomarkers into a single model will substantially improve the diagnostic value [11]. Moreover, microRNAs (miRNAs) were identified as short non‐coding RNAs with a length of approximately 20‐25 nucleotides, which exert significant impacts on numerous biological processes [12]. MiRNAs might control gene expression in the post‐transcriptional levels by pairing with target mRNAs at the 3′ untranslated region (3′ UTR) [13]. A substantial number of investigations have revealed that miRNAs are involved in the progression of AS. For example, it has been reported that miRNA-33 modulated the macrophage autophagy in AS [14]. MiRNA-181b regulated AS and aneurysms by controlling the expression of TIMP-3 and Elastin [15]. However, the miRNA expression-based signatures for the diagnosis of AS were still limited.

In this study, we aimed to identify the miRNA-based diagnostic signature and construct the predicting model for the diagnosis of AS by combining bioinformatics analysis and machine learning, which will benefit the development of the early diagnosis strategy of AS.

## Materials and methods

### Data collection

The miRNA microarray data of GSE59421 [16], containing 33 blood samples of patients with AS and 63 healthy control blood samples, and the mRNA microarray data of GSE20129 [17], including 57 peripheral blood samples from patients with AS and 78 peripheral blood samples from healthy cases, were obtained from the Gene Expression Omnibus (GEO, https://www.ncbi.nlm.nih.gov/geo/) database. The expression value of miRNA microarray data of GSE59421 was detected using the Agilent-021827 Human miRNA Microarray (V3) (miRBase release 12.0 miRNA ID version). The expression value of mRNA microarray data of GSE20129 was detected using the Illumina humanRef-8 v2.0 expression beadchip. The clinical information of samples was shown in Additional file 6: Table S1. Besides, the bioinformatics workflow was shown in Additional file 1: Fig. S1.

### Weighted gene co-expression network analysis (WGCNA)

The weighted gene co-expression network analysis (WGCNA) was performed by the *WGCNA R* package in the samples [18]. The hierarchical cluster was conducted according to the miRNA expression of the samples, and the miRNA with higher similarity in expression were identified in modules using the dynamic cut tree method. The characteristic gene (Module Eigengene, ME) value of each module and the correlation coefficient of the ME value with the phenotype, including the sample type (AS or not) and age, were calculated.

### Functional enrichment analysis

After the potential miRNAs were screened from WGCNA analysis, we further predicted the genes targeted by these miRNAs using the miRTarBase database (Release 7.0: Sept. 15, 2017 mirtarbase.mbc.nctu.edu.tw). Then, Gene Ontology (GO) and Kyoto Encyclopedia of Genes and Genomes (KEGG) [19] pathway enrichment analysis were performed by using *clusterProfiler* package of *R* software [20]. *P* < 0.05 was regarded as statistically significant.

### Protein–protein interaction analysis

The function and interactions of proteins were analyzed and predicted by The STRING database (https://string-db.org/,version 11.0) [21]. Besides, the protein–protein interaction analysis (PPI) was visualized by Cytoscape software (version 3.7.2) [22].

### Logistic regression models

The logistic regression models were constructed, in which the expression value of identified miRNAs was considered as predictive variables, and the sample type (AS or not) was considered as a binary responsive variable. The samples from GSE59421 and GSE20129 were used for the construction of the multivariable logistic regression model using *glm* of *R*, followed by the stepwise regression method to filter out the significant variables finally included in the model with *P* < 0.05 as the threshold. The threefold cross-validation was performed in the GSE59421 cohort to validate the accuracy of the logistic regression models by *caret* function of R language (https://CRAN.R-project.org/package=caret). The receiver operating characteristic (ROC) curves were generated to evaluate the sensitivity and specificity of the logistic regression models, and the area under the curve (AUC) was calculated to assess the accuracy of the models.

## Results

### MiRNAs and mRNAs related to AS are identified by WGCNA.

**Fig. 1:**
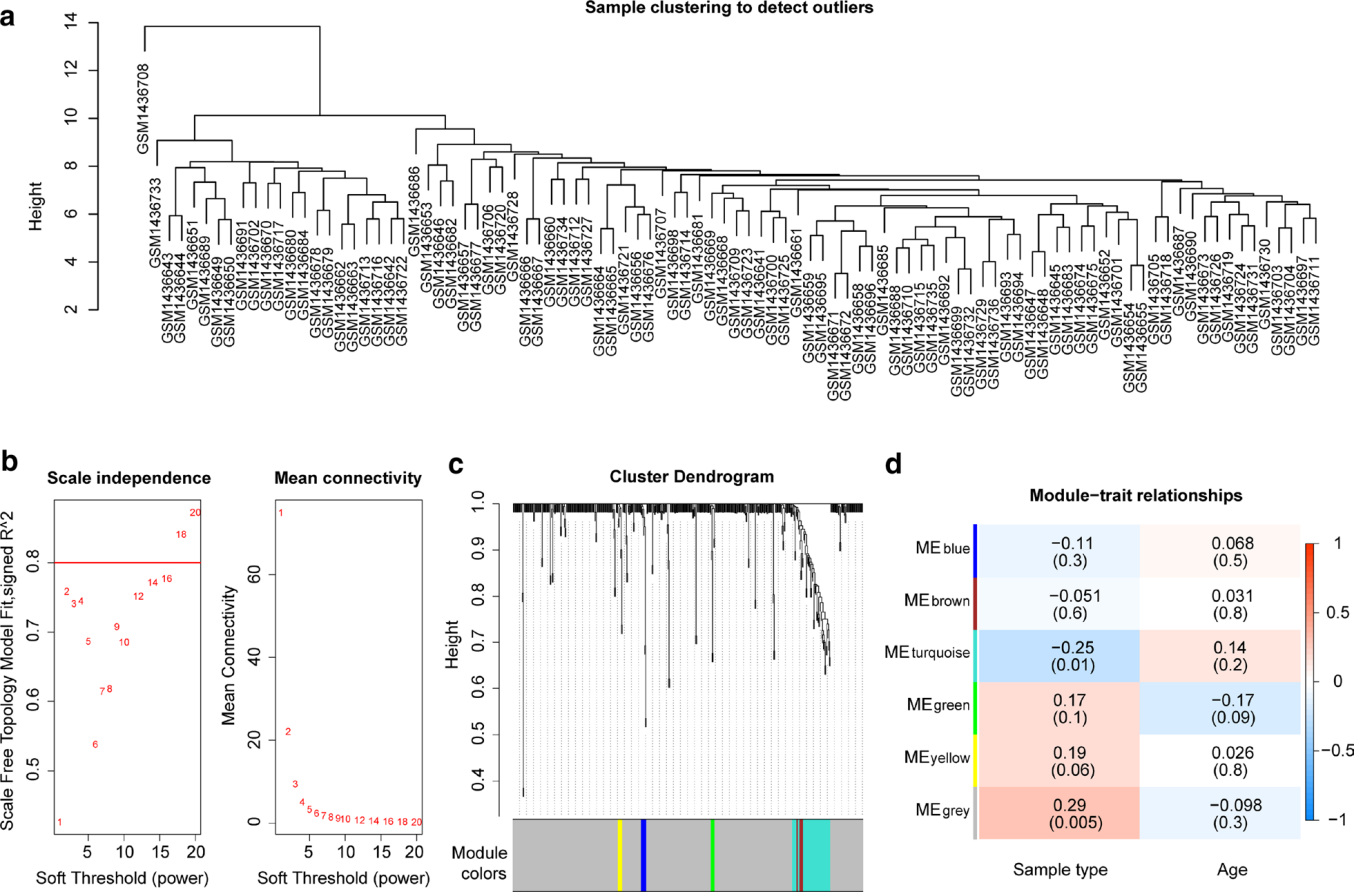
WGCNA analysis miRNA microarray data of GSE59421. **a** Schematic diagram of clustering analysis was shown. **b** Schematic diagram of soft threshold screening was shown, in which the red line in the figure was the correlation coefficient, and the first point above the red line was the soft threshold β = 18. **c** Schematic diagram of gene module clustering was shown, in which each color represented a module, the genes in the gray module were genes that were not clustered into any module. **d** The heatmap of the correlation between the gene module and the phenotype was shown, in which the color of red and blue represented the phenotypic correlation

**Fig. 2:**
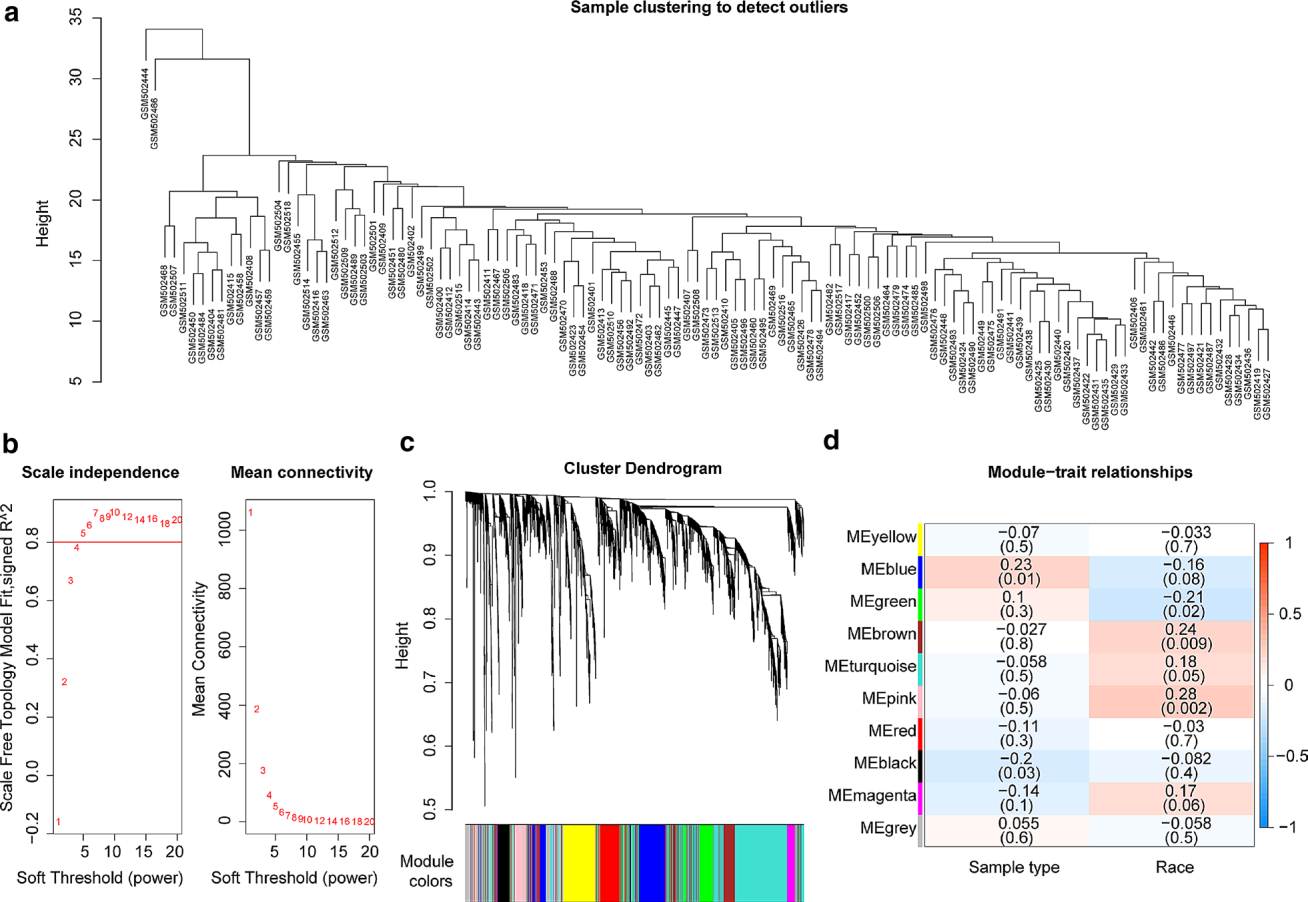
WGCNA analysis mRNA microarray data of GSE20129. **a** Schematic diagram of clustering analysis was shown. **b** Schematic diagram of soft threshold screening was shown, in which the red line in the figure was the correlation coefficient, and the first point above the red line was the soft threshold β = 5. **c** Schematic diagram of gene module clustering was shown, in which each color represented a module, the genes in the gray module were genes that were not clustered into any module. **d** The heatmap of the correlation between the gene module and the phenotype was shown, in which the color of red and blue represented the phenotypic correlation

To explore the potential miRNAs and mRNAs that were correlated with AS, GSE59421 and GSE20129 were normalized to minimize the batch deviation of gene expression intensity (Additional file 2: Fig. S2A and B). The cluster analysis was performed based on the miRNA microarray data of GSE59421, in which an outlier sample was excluded in the subsequent analysis (Fig. 1a). Besides, the power value of β = 18 (scale-free R^2^ = 0.80) was selected as the soft-thresholding parameter to construct a scale-free network (Fig. 1b). A total of 6 modules were identified through the average linkage hierarchical clustering (Fig. 1c). The correlation of the modules with the sample type (AS or not) and age was analyzed, in which the turquoise module contained 42 miRNAs presented the highest association with the sample type (*P* = 0.01) (Fig. 1d). The cluster analysis was also conducted based on the mRNA microarray data of 119 samples from GSE20129 detected by Illumina humanRef-8 v2.0 expression beadchip platforms, in which two outlier samples were excluded in the subsequent analysis (Fig. 2a). The power value of β = 5 (scale-free R^2^ = 0.80) was selected as the soft-thresholding parameter to construct a scale-free network (Fig. 2b). A total of 10 modules were identified through the average linkage hierarchical clustering (Fig. 2c). The correlation of the modules with the sample type and age was analyzed, in which the blue module contained 532 genes showed the highest association with the sample type (*P* = 0.01) (Fig. 2d). Together these data suggest that the 42 miRNAs and 532 genes determined from the WGCNA might be closely correlated with AS.

### Functional enrichment analysis identifies 12 miRNAs potentially critical for patients with AS

**Fig. 3:**
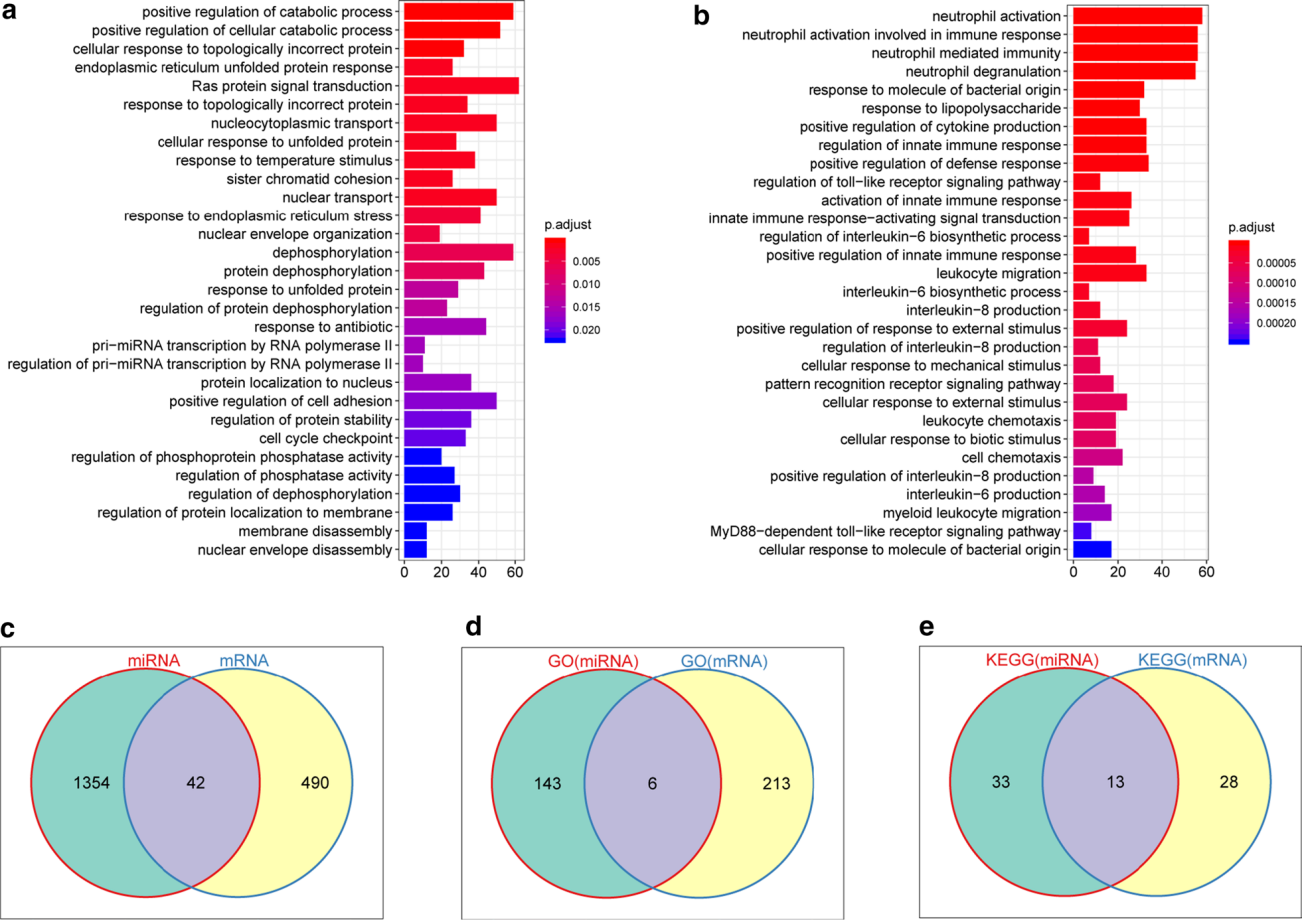
Functional enrichment analysis. **a** The top 30 significant cellular processes based on the 1396 genes targeted by the 42 miRNAs were presented by GO enrichment scatter plot. The y-axis was the name of GO terms, and the x-axis was the gene ratio; The size of the dot revealed the number of genes. **b** The top 30 significant cellular processes based on the 532 genes in the blue module were presented by GO enrichment scatter plot. **c**–**e** The overlap of genes, GO terms, and KEGG pathways between the 1396 genes targeted by the 42 miRNAs in the turquoise module and the 532 genes in the blue module were presented in the Venn diagram

The turquoise miRNA module contained 42 miRNAs and the targeted-gene prediction analysis identified 1396 potential targeted genes for these miRNAs (Additional file 7: Table S2) by the miRTarBase database (Release 7.0: Sept. 15, 2017 mirtarbase.mbc.nctu.edu.tw). For primary comprehensions of the identified 1396 genes, GO and KEGG pathway enrichment analysis were performed. Multiple GO terms, such as positive regulation of catabolic process and response to topologically incorrect protein, and KEGG pathways, for instance, cellular senescence and fluid shear stress and atherosclerosis, were revealed based on the 1396 genes (Additional file 8: Table S3), in which the top 30 remarkable GO terms and KEGG pathways were demonstrated (Fig. 3a and Additional file 3: Fig. S3A). Meanwhile, GO and KEGG pathway analysis were conducted based on the 532 mRNAs of the blue module. Several GO terms, such as response to lipopolysaccharide, and KEGG pathways, for instance, fluid shear stress and atherosclerosis, were enriched based on the 532 genes (Additional file 9: Table S4), in which the top 30 remarkable GO terms and KEGG pathways were demonstrated (Fig. 3B and Additional file 3: Fig. S3B).

More importantly, 42 overlapped genes among the 532 genes in the blue module and 1396 targeted genes of the 42 miRNAs were identified (Fig. 3c), which were targeted by 12 of the 42 miRNAs. In addition, the functional enrichment analysis identified 6 overlapped GO terms (Fig. 3d) and 13 overlapped KEGG Pathways (Fig. 3e) between the 1396 genes targeted by the 42 miRNAs in the turquoise module and the 532 genes in the blue module (Additional file 10: Table S5). Together these data indicate that the identified 12 miRNAs are potentially critical for patients with AS.

### PPI network construction identifies the genes

Based on the STRING database, the PPI network was constructed for 42 genes, the minimum required interaction score > 0.4 was used as the threshold to screen the interaction protein, and then the PPI network was visualized by the Cytoscape software, as shown in Additional file 4: Fig. S4. There were 14 nodes and 11 edges in total in Additional file 4: Fig. S4, and a node represented a gene, and an edge represented the interaction between two nodes.

### Establishment, verification, and evaluation of the logistic regression model based on two miRNAs

**Fig. 4:**
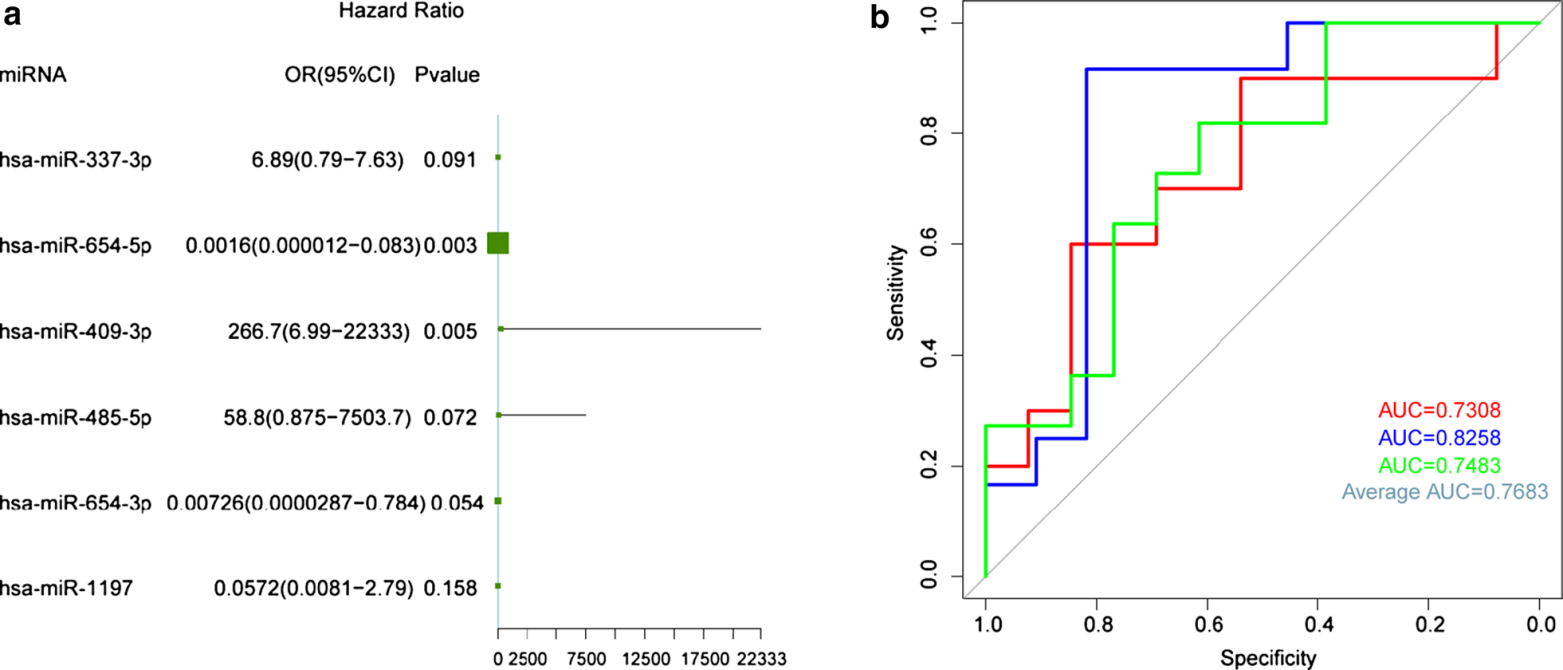
Logistic regression model construction. **a** The forest plot of the 6 identified miRNAs in the model was shown. **b** The ROC curve of the logistic regression model was shown. The x-axis represented the specificity of the negative positive rate (false positive rate FPR), and the y-axis represented the sensitivity of the true positive rate (TRR)

Next, with the untreated patients as control group, a logistic regression model was constructed in the samples of GSE59421 and GSE20129 based on the identified 12 miRNAs and among them, 6 miRNAs were further identified by the stepwise regression analysis for the further analysis, including hsa-miR-337-3p, hsa-miR-654-5p, hsa-miR-409-3p, hsa-miR-485-5p, hsa-miR-654-3p, and hsa-miR-1197. Then, these 6 miRNAs as the variables were incorporated into multivariate logistic regression analysis, in which the *P* values of hsa-miR-654-5p and hsa-miR-409-3p were less than 0.05 (Fig. 4a), indicating that these miRNAs might be significantly related to the occurrence of AS. Besides, the logistic regression model based on the 2 miRNAs was reconstructed, and it was confirmed to conform to the normal distribution (Additional file 5: Fig. S5A). What’s more, there was a good linear correlation between the predictor variable and responsive variable in the model (Additional file 5: Fig. S5B), and there were no extreme points that significantly affected the accuracy in the model (Additional file 5: Fig. S5C).

Next, we used the threefold cross-validation method to evaluate the reliability of the model. The samples from GSE59421 were randomly divided into training set and verification set, which was used for constructing the logistic regression model and verification of the model, respectively. Our data showed that the AUCs of the three verification sets in the logistic model constructed by the threefold cross-validation method were 0.7308, 0.8258, and 0.7483 with an average AUC of 0.7683 (Fig. 4b), suggesting that the logistic regression model based on these two miRNAs could reliably distinguish the patients with AS and healthy cases, and these 2 miRNAs could be used as potential biomarkers for the diagnosis of patients with AS.

## Discussion

AS is a complex multifactorial disease that, despite advances in lifestyle management and drug therapy, remains to be the major cause of high morbidity and mortality rates from cardiovascular diseases in industrialized countries [23, 24]. Therefore, it is urgent to seek reliable diagnostic biomarkers and effective treatment alternatives to reduce its burden [25]. MiRNAs have received most of the attention over the last decades in particular for their role in tempering gene expression [13]. An increasing number of studies have highlighted the importance of miRNAs in the development and progression of AS [26]. Recently, it was shown that miRNAs exert their role in the pathophysiology of AS via the regulation of AS -prone genes as well as their impact in regulating post-transcriptional gene expression [27]. In this study, a total of 42 miRNAs and 532 genes showed the highest association with AS in the WGCNA. Moreover, it has been identified that catabolic process, neutrophil activation, and TNF signaling are involved in the modulation of the development of AS [28–30]. Our GO and KEGG pathway analysis based on the 1396 potential targeted genes of the 42 miRNAs and the identified 532 genes in the WGCNA presented multiple cellular processes, such as positive regulation of catabolic process, Renal cell carcinoma, neutrophil activation, and TNF signaling pathway. Our data were consistent with the previous study that the positive regulation of catabolic process, neutrophil activation, and TNF signaling pathway participated in the modulation of AS. More importantly, overlap analysis identified 42 overlapped genes among the 532 genes in the blue module and 1396 targeted genes of the 42 miRNAs, in which these 42 overlapped genes were targeted by 12 miRNAs. These data suggest that these 12 miRNAs are potentially critical for patients with AS.

The pathogenesis of AS is complicated, and it has been identified that miRNAs are involved in the development of AS. For example, miR-654-3p is involved in the lncRNA ZFAS1-mediated inflammation responses in AS by targeting ADAM10 and RAB22A [31]. Meanwhile, miR-212, miRNA-216a, and miRNA-377 are considered as the potential biomarkers for the diagnosis of AS [32, 33]. In the present study, a total of 2 miRNAs, hsa-miR-654-5p and hsa-miR-409-3p were identified and the threefold cross-validation method showed that the AUC of logistic regression model based on these 2 miRNAs was 0.7308, 0.8258, and 0.7483 with an average AUC of 0.7683. As indicated above, hsa-miR-654-3p among our identified miRNAs have been reported to associate with AS. Our data, along with the previous reports further suggest that our logistic regression model can reliably predict the diagnosis of patients with AS.

Moreover, the functional enrichment analysis results illustrated that the identified genes significantly related to fluid shear stress and atherosclerosis pathway, indicating that the results might reliable. Besides, the other two pathways screened out, response to topologically incorrect protein, and response to lipopolysaccharide, were markedly associated with genes and atherosclerosis. Based on the topological data analysis of quantitative whole-heart coronary plaque characteristics, recent research suggested that varies patients has distinct plaque dynamics and clinical outcomes [34]. In addition, several studies revealed that microbiota could influence the atherosclerosis by regulating lipopolysaccharide production and intestinal homeostasis [35, 36]. The above researches were consistent with our results. In the future study, we will explore the regulation mechanism of critical miRNA.

In conclusion, this study identified a total of 2 miRNAs, including hsa-miR-654-5p and hsa-miR-409-3p, are identified as the potentially critical biomarkers for AS. The logistic regression model based on the identified 2 miRNAs could reliably distinguish the AS patients from normal cases. Our finding presents new insights into the miRNA-based signatures for AS and provide valuable predictive model, benefiting the diagnosis of AS patients.

## Supplementary Information

**Additional file 1: Figure S1**. The bioinformatics workflow of this study.

**Additional file 2: Figure S2**. Data standardization. (A) The distribution of miRNA expression value in each sample of GSE59421 data after normalization was shown. The x-axis was the sample, and the y-axis was the miRNA expression value. (B) The distribution of mRNA expression value in each sample of GSE20129 data after normalization was shown. The x-axis was the sample, and the y-axis was the mRNA expression value.

**Additional file 3: Figure S3**. Kyoto Encyclopedia of Genes and Genomes (KEGG) pathway analysis. (A) The top 30 significant KEGG pathways based on the 1396 genes targeted by the 42 miRNAs. (B) The top 30 significant KEGG pathways based on the 532 genes in the blue module.

**Additional file 4: Figure S4**. Protein-protein interaction analysis for the 42 overlapped genes among the 532 genes in the blue module and 1396 targeted genes of the 42 miRNAs.

**Additional file 5: Figure S5**. Logistic model diagnosis diagram. (A) A normal Q-Q graph was shown. (B) The component plus residual plot based on the identified 2 miRNAs in the model was shown. (C) The Residuals vs Leverage was shown.

**Additional file 6: Table S1**. The clinical information of samples in this study.

**Additional file 7: Table S2**. Predicted target genes of the 42 identified miRNAs in the WGCNA.

**Additional file 8: Table S3**. The information of KEGG analysis based on the identified 1396 genes.

**Additional file 9: Table S4**. The information of KEGG analysis based on the identified 532 genes.

**Additional file 10: Table S5**. The overlap KEGG Pathways between the 1396 genes targeted by the 42 miRNAs in the turquoise module and the 532 genes in the blue module.

## Data Availability

The data were obtained from the Gene Expression Omnibus (GEO, https://www.ncbi.nlm.nih.gov/geo/) database.

## Abbreviations

AS: Atherosclerosis
WGCNA: Weighted gene co-expression network analysis
miRNAs: MicroRNAs
GO: Gene ontology
KEGG: Kyoto encyclopedia of genes and genomes
AUC: Area under the curve
ROC: Receiver operating characteristic
