# Supplementary figures and images for: A 2 miRNAs-based signature for the diagnosis of atherosclerosis
Source: BMC Cardiovasc Disord. 2021 Mar 24;21:150. doi: 10.1186/s12872-021-01960-4 (PMC7988968; doi:10.1186/s12872-021-01960-4)

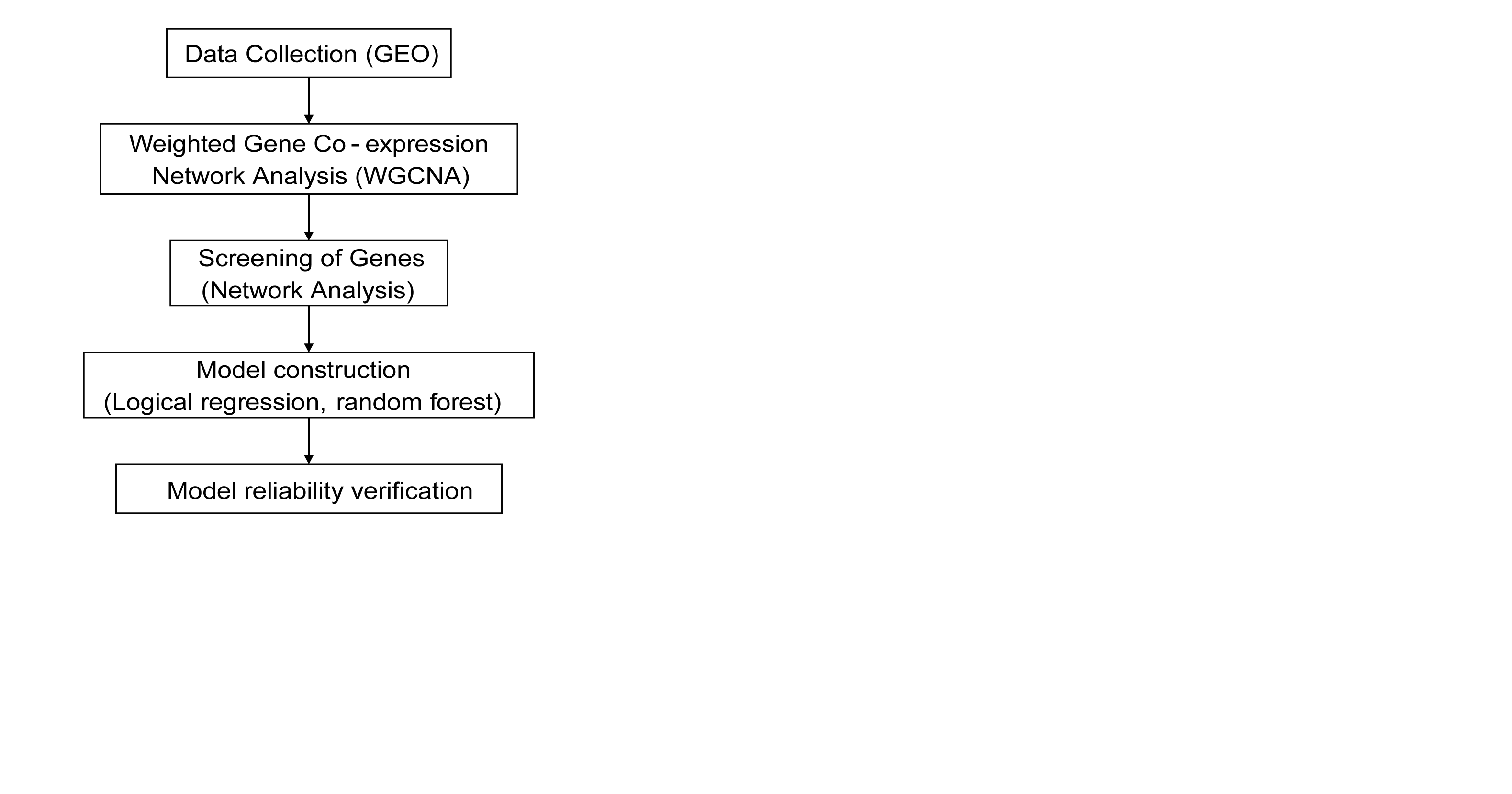

Supplement: Supplementary file 1 — Additional file 1: Figure S1. The bioinformatics workflow of this study. [file 12872_2021_1960_MOESM1_ESM.tif]

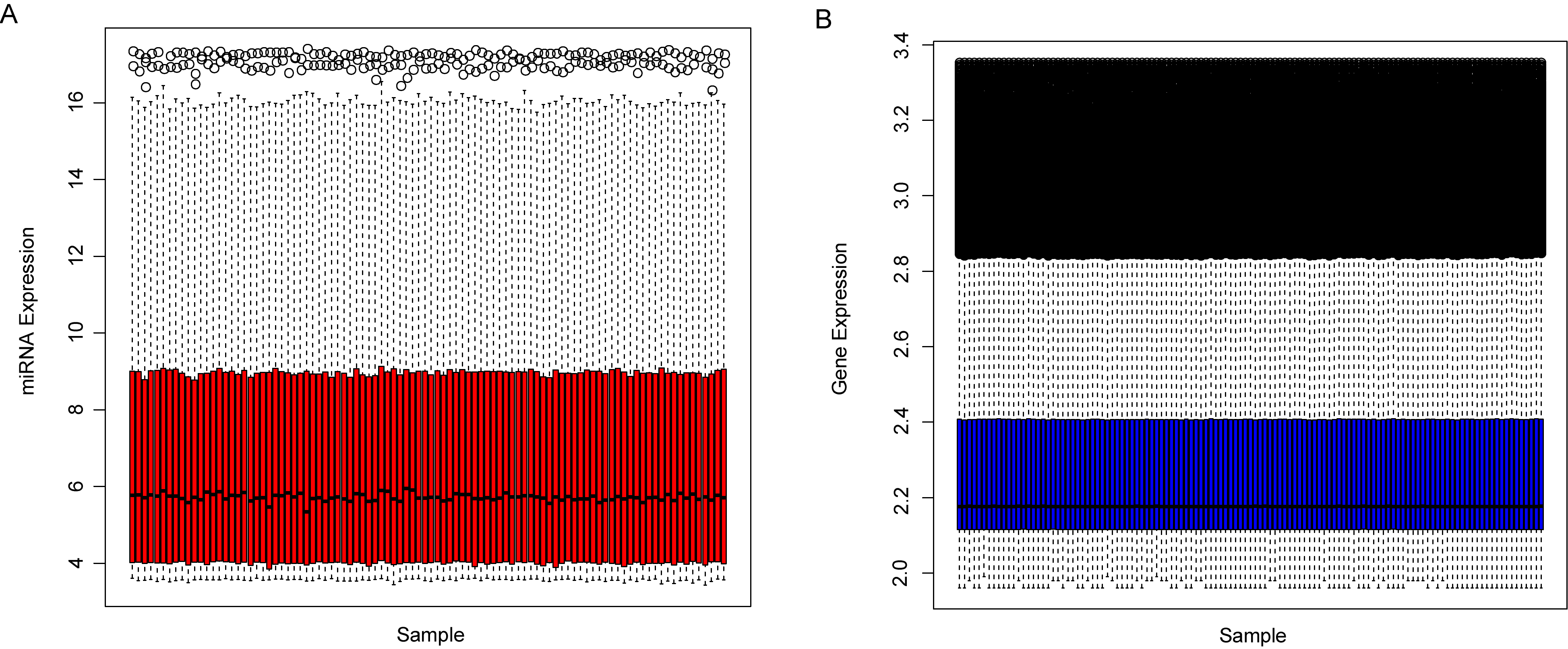

Supplement: Supplementary file 2 — Additional file 2: Figure S2. Data standardization. (A) The distribution of miRNA expression value in each sample of GSE59421 data after normalization was shown. The x-axis was the sample, and the y-axis was the miRNA expression value. (B) The distribution of mRNA expression value in each sample of GSE20129 data after normalization was shown. The x-axis was the sample, and the y-axis was the mRNA expression value. [file 12872_2021_1960_MOESM2_ESM.tif]

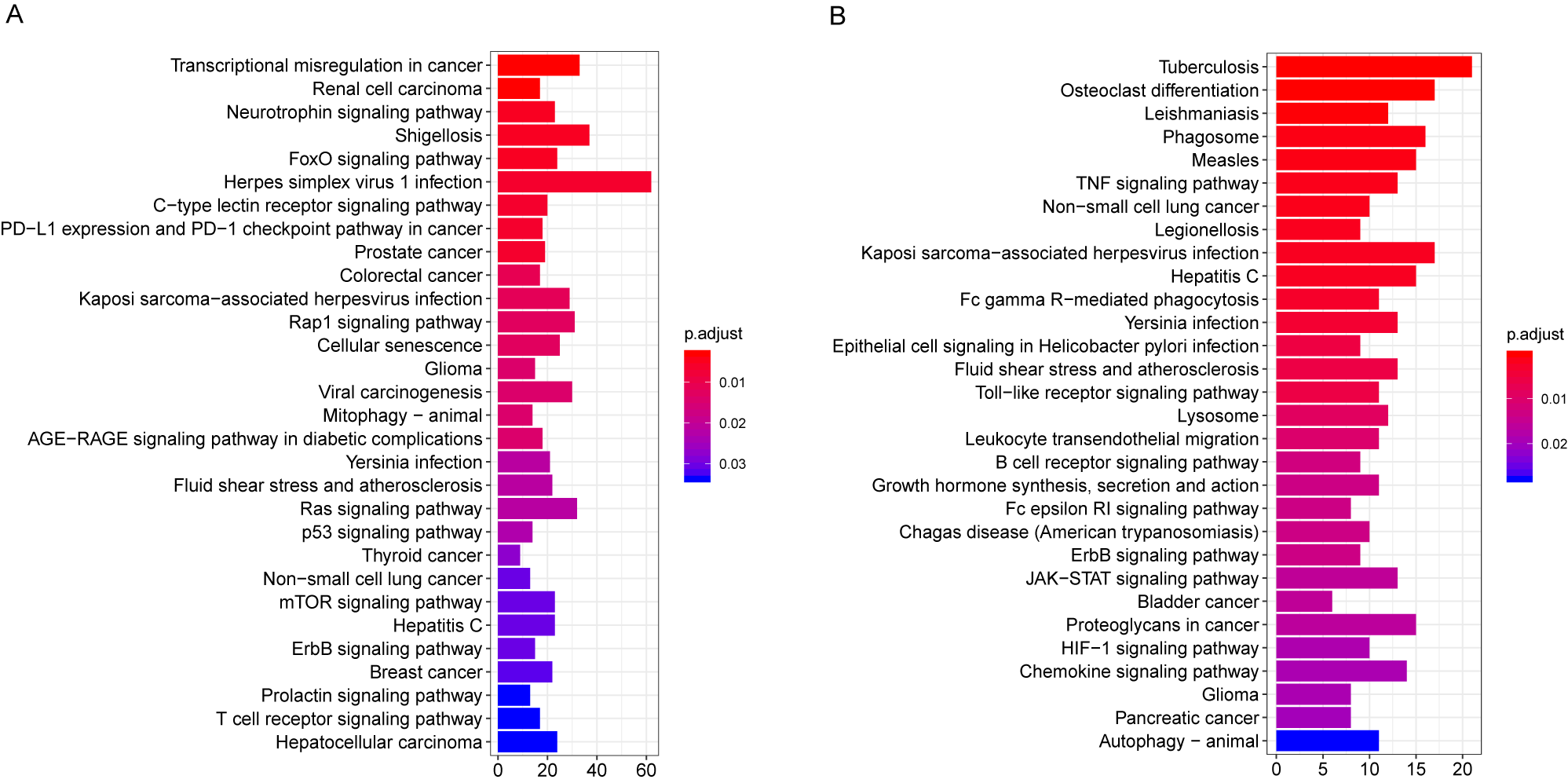

Supplement: Supplementary file 3 — Additional file 3: Figure S3. Kyoto Encyclopedia of Genes and Genomes (KEGG) pathway analysis. (A) The top 30 significant KEGG pathways based on the 1396 genes targeted by the 42 miRNAs. (B) The top 30 significant KEGG pathways based on the 532 genes in the blue module. [file 12872_2021_1960_MOESM3_ESM.tif]

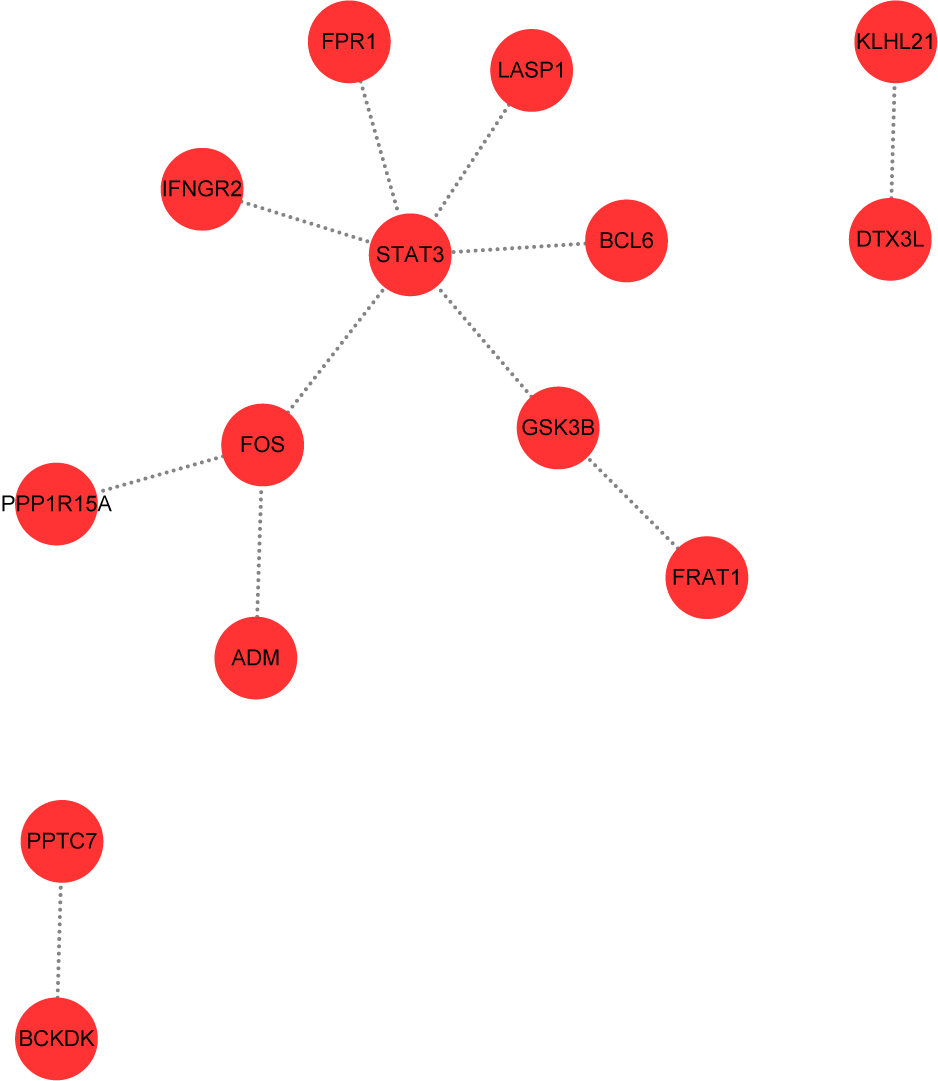

Supplement: Supplementary file 4 — Additional file 4: Figure S4. Protein-protein interaction analysis for the 42 overlapped genes among the 532 genes in the blue module and 1396 targeted genes of the 42 miRNAs. [file 12872_2021_1960_MOESM4_ESM.tif]

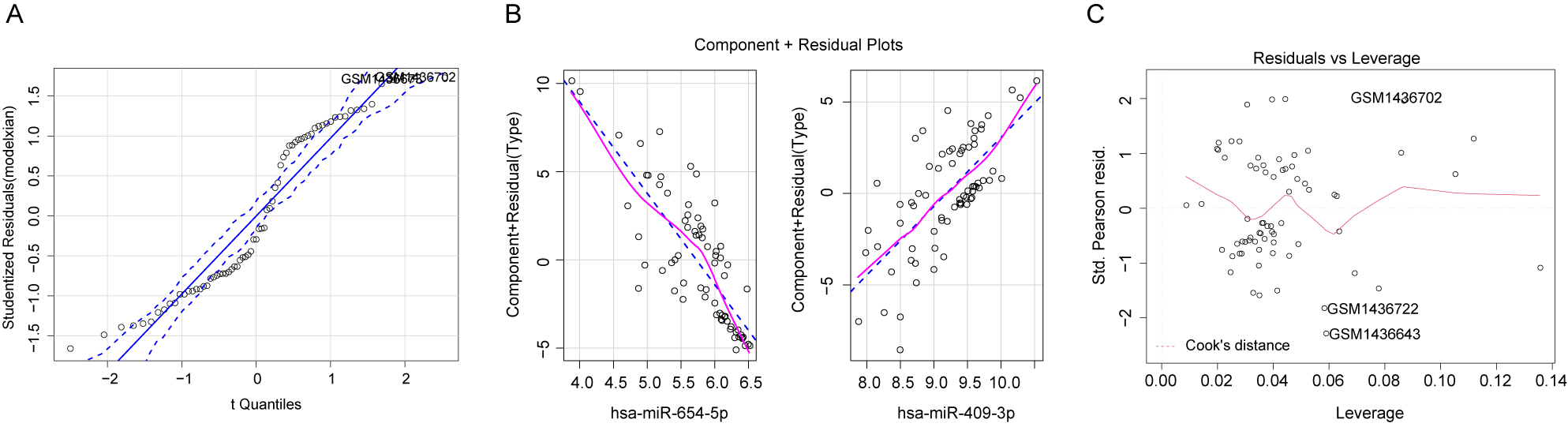

Supplement: Supplementary file 5 — Additional file 5: Figure S5. Logistic model diagnosis diagram. (A) A normal Q-Q graph was shown. (B) The component plus residual plot based on the identified 2 miRNAs in the model was shown. (C) The Residuals vs Leverage was shown. [file 12872_2021_1960_MOESM5_ESM.tif]
